# Automatic Assessment of the Type and Intensity of Agitated Hand Movements

**DOI:** 10.1007/s41666-022-00120-3

**Published:** 2022-09-23

**Authors:** Fiona Marshall, Shuai Zhang, Bryan W. Scotney

**Affiliations:** School of Computing, University of Ulster, Shore Road, Newtownabbey, BT37 0QB Northern Ireland UK

**Keywords:** Pervasive computing, Agitation, Pattern recognition, Skeletal keypoint models, Behaviour observation

## Abstract

With increasing numbers of people living with dementia, there is growing interest in the automatic monitoring of agitation. Current assessments rely on carer observations within a framework of behavioural scales. Automatic monitoring of agitation can supplement existing assessments, providing carers and clinicians with a greater understanding of the causes and extent of agitation. Despite agitation frequently manifesting in repetitive hand movements, the automatic assessment of repetitive hand movements remains a sparsely researched field. Monitoring hand movements is problematic due to the subtle differences between different types of hand movements and variations in how they can be carried out; the lack of training data creates additional challenges. This paper proposes a novel approach to assess the type and intensity of repetitive hand movements using skeletal model data derived from video. We introduce a video-based dataset of five repetitive hand movements symptomatic of agitation. Using skeletal keypoint locations extracted from video, we demonstrate a system to recognise repetitive hand movements using discriminative poses. By first learning characteristics of the movement, our system can accurately identify changes in the intensity of repetitive movements. Wide inter-subject variation in agitated behaviours suggests the benefit of personalising the recognition model with some end-user information. Our results suggest that data captured using a single conventional RGB video camera can be used to automatically monitor agitated hand movements of sedentary patients.

## Introduction

Agitated and repetitive behaviours are some of the most common and demanding symptoms of dementia [1]. Agitation is distressing for both patient and caregiver, challenging to manage, and can lead to physical injury. Agitation is assessed using tools such as the neuropsychiatric inventory [2] and the Cohen Mansfield Agitation Index [3], which are based upon the prevalence of agitated behaviours. As assessments rely on a carer’s recall of incidents of agitation over several weeks [4], they may be subjective and slight changes in behaviour may go unnoticed. Due to the difficulty in providing accurate metrics, most assessments monitor the frequency but not the severity of agitated episodes. Small, agitated movements can be indicators of potential danger such as an unsteady patient trying to rise from their seat, increased frustration, or anxiety. Monitoring agitation can lead to a better understanding of the patient’s condition and enable more effective care [5]. Automatic monitoring of repetitive behaviours could provide consistent, objective, and continuous monitoring of agitation. An automatic system would provide accurate metrics for assessing agitation, whilst relieving pressure on carers. Despite potential advantages, there has been little research into the automatic monitoring of repetitive behaviours. Moreover, repetitive hand movements have been largely overlooked [1], with research focusing on gross body movements such as pacing, kicking, or hitting [6, 7]. Meanwhile, models to recognise hand movements have focused on emblematic hand gestures for sign language interpretation or human–computer interaction [8]. Recognition of repetitive hand movements is essential for distinguishing between normal and beneficial behaviours such as self-soothing actions, and abnormal and potentially harmful behaviours like scratching.

RGB cameras are widely available and provide a rich source of data. Video-based approaches are particularly suitable for patients who are mainly sedentary. For many patients, a video camera directed towards a favourite chair, perhaps situated above a television screen, offers the potential to capture rich data over significant periods. In addition to capturing the whole body, video can provide detailed information about hand movement. Using opensource algorithms to extract skeletal keypoint locations, a person can be represented as a “stickman.” Reducing video data to a sequence of skeletal poses provides informative features ideal for monitoring behaviour. Skeletal models can protect patient privacy by removing the need to retain images.

This research establishes the potential for automatic classification of repetitive hand movements and automatic assessment of the intensity of repetitive hand movements using skeletal keypoints extracted from video. We conjecture that first recognising the type of behaviour will aid assessing intensity. The feasibility of using video-based keypoints to monitor agitated hand movements is explored using a small dataset containing video in which healthy volunteers demonstrate repetitive hand movements according to the expected norms of agitated behaviour. Due to the size of the dataset, methods are confined to those suitable for small datasets. Five types of repetitive hand movements are studied: picking, scratching, rubbing, wringing, and clapping. The first four movements are indicative of agitated behaviours observed in people living with dementia [3]; clapping is included as a benchmark activity. The paper is organised as follows: Section 2 contains a summary of previous related research. In Section 3, details of data collection, data cleaning, classification of the type of movement, and assessment of the intensity of repetitive movements are given. Section 4 reports results for classifying and assessing of the intensity of unknown repetitive hand movements from a test dataset. Section 5 contains a discussion of results and the future direction of research.

## Related Research

A range of sensors has been employed to assess repetitive behaviours, including accelerometers [9–11], depth sensors [6, 7], and video [12, 13]. Whilst depth sensors and video cameras have been widely used for activity and gesture recognition, the high dimensionality of their output can result in computationally expensive algorithms requiring large amounts of training data. However, the dimensionality of the data can be greatly reduced by representing a person’s movement by a sequence of skeletal keypoint locations whilst still retaining essential information [14]. Hand and body keypoints can be extracted from data collected with a 3D sensor or video camera. The low cost and availability of the Kinect 3D sensor [15] has led to much interest in skeleton-based action recognition, although Kinect does not provide hand keypoints. Leap Motion [16] and Intel RealSense [17] provide 3D sensors able to locate hand keypoints. An advantage of video cameras over depth sensors is that they have a much larger range; both 3D hand keypoint detectors only capture keypoints within 60 cm of the sensor. Several opensource algorithms extract 2D body keypoints from video including OpenPose [18] and MediaPipe [19]. Body keypoints can be extracted in real-time, making video-based keypoints suitable for patient monitoring and providing alerts. Both systems provide hand keypoint detection, enabling the recognition of hand movements. Keypoints obtained from video have been shown to be as effective for activity recognition as 3D keypoints [20].

The detection, recognition, and measurement of the intensity of repetitive movements are fundamental components for monitoring agitated behaviours. The first component, the detection of repetitive movements, is the most extensively researched. However, most studies have focused on showing the correlation between motor activity and clinical agitation scores. Studies using accelerometers have shown a correlation between disruptive sleep patterns and night-time incontinence [21], activity levels [21], aggressive behaviour [22] and differing circadian patterns [23]. We are aware of a single system that detects agitation and alerts carers to enable intervention: BESI [9] uses recurrent neural networks (RNN) to learn patterns of behaviours from data obtained from smartwatches and provide real-time notification of agitated behaviours. Whilst an accelerometer is useful for continuous monitoring, unlike a video sensor, it is limited to capturing motion from a single point of the body, restricting its ability to distinguish between types of agitation. Video and depth sensors capture rich information that can be used for the detection of repetitive behaviours. Kicking has been identified by tracking changes in foot location [24], and pacing detected using signal correlation to analyse a person’s trajectory [13]. Whilst both systems used background subtraction to locate body parts, recent keypoint extraction approaches could enable the techniques to be applied more efficiently. More recently, skeletal keypoint locations obtained from a depth sensor have been used to detect repetitive behaviours in autistic children [25].

Recognising the type of repetitive movement is the second component of agitation monitoring. A study comparing the effectiveness of accelerometers and 3D keypoint models for agitation recognition found that data obtained from accelerometers were computationally more efficient but slightly less accurate than skeletal pose data [6]. Skeleton-based activity recognition is a well-developed field whose models can be adapted to recognise repetitive behaviours. Although deep learning is widely used for activity recognition, it requires large amounts of training data and can be resource and memory intensive. Restricted by small datasets, models classifying repetitive behaviours from keypoint data have used traditional machine learning approaches such as support vector machines (SVM) [7], rotation forest [6], dynamic time warping (DTW) [25] and nearest neighbour [26]. Although hand gesture classification models have been used for human–computer interaction and sign-language interpretation [8], we are not aware of any studies that have explored the classification of agitated hand movements.

The third component for monitoring agitation is measuring the intensity of agitated episodes. The intensity of a movement is defined as the frequency of repetitions during a specified period. IMUs [10, 11, 27] and video-based keypoints [28] have been used for gait analysis, whilst systems to automatically measure the intensity of repetitive hand movements in people with Bradykinesia have used video-based keypoints [12, 29] and electromagnetic sensors attached to the fingers [30, 31].

This study demonstrates the potential for using video-based keypoints to identify the type and assess the intensity of agitated hand movements. Reducing a video image to a sequence of keypoints significantly reduces the number of features in each frame whilst retaining important pose information, reducing computational cost. However, recognising agitated hand movements from skeletal poses remains challenging due to the number of hand keypoints and the range of possible hand movements.

## Method

**Fig. 1:**
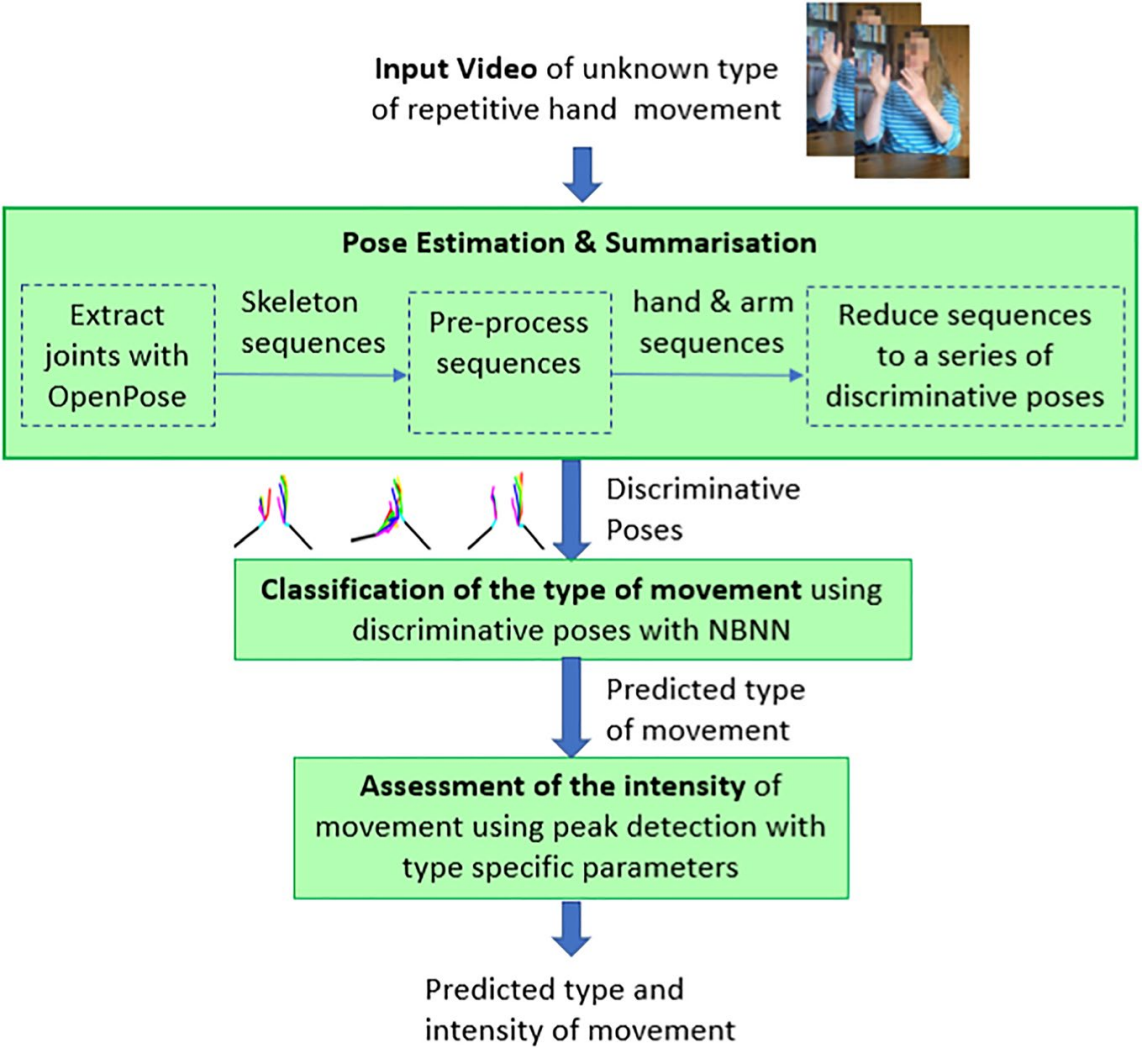
System for automatically assessing both the type and the intensity of repetitive hand movements. Given a video sequence, keypoints are extracted, and the hand movement is summarised by discriminative poses. The type of movement is classified using NBNN. The intensity of the movement is then assessed using distance measures based on the predicted type of movement

This section describes a system for automatically assessing both the type and intensity of unknown hand movements. Figure 1 illustrates the system’s pipeline, which can be divided into three stages: pose estimation and summarisation (Section 3.2), classification of the type of movement (Section 3.3), and assessment of the intensity of movement (Section 3.4). The capacity of the combined system to automatically assess the type and intensity of unknown hand movements by first classifying the type of hand movement, and thereby selecting appropriate parameters for assessing the intensity of movement is tested in Section 4.

### Data Collection

Most hand datasets contain hand gestures used for human–computer interaction [32, 33] or sign language recognition [34, 35], where sequences are restricted to gestures rather than spontaneous or repetitive movements. Moreover, many of the datasets contain only single hand poses. As there is no suitable open dataset available, a novel video-based dataset of five repetitive hand movements was collected. The study was approved by the relevant Ulster University Faculty Research Ethics Filter Committee.

Twenty healthy participants were recorded by an RGB camera demonstrating five hand movements: *clapping*, *picking arm*, *scratching arm*, *hand wringing*, and *rubbing an object*. The dataset was split into training and test sets. Data from fifteen participants were used for the training and validation of classifiers to identify the type of movement and to learn parameters for measuring the intensity of repetitive movements. Data from the remaining five participants were reserved for testing the robustness of the system with new subjects. The dataset was recorded by participants in their own homes, creating a varied and demanding dataset that is representative of home or care settings. Variations included participant position (standing or seated), dominant hand (the hand which moves the most), hand position (the location and orientation of the hand in relation to the body), and whether one or both hands move. Similarities between different types of hand movements and variations in the intensity of movement add to the complexity of the dataset. Each movement was performed for 30 s at four different intensities: *slow*, *medium*, *medium-fast*, and *fast*, creating a dataset of 400 sequences*.* A metronome set correspondingly at 30, 50, 70, and 90 repetitions per minute (rpm) was used to pace the hand movements. Most videos were recorded at a speed of 30 frames per second; those not were up- or down-sampled to this rate.

### Pose Estimation and Summarisation

**Fig. 2:**
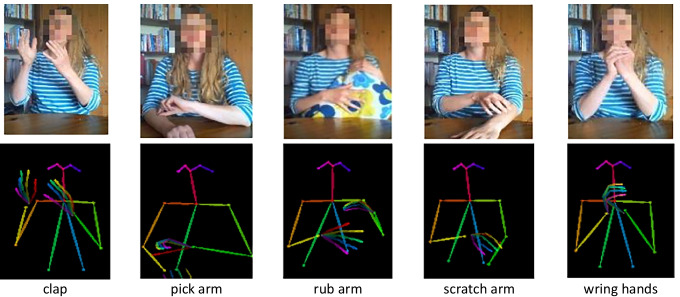
Five repetitive movements were recorded, and keypoint positions extracted using OpenPose

Each 30-second training data sequence was divided into four non-overlapping 6-second (180 frame) sequences. Three seconds of data were discarded at both ends of the original sequence to remove any different movements from the start and end of a sequence, creating a labelled dataset of 1200 6-s sequences. OpenPose [36] is used to represent the body and hands as a set of keypoint locations, as show in Fig. 2. Both the classification and the intensity models use features created from hand and arm keypoints; the advantages of combining hand and arm keypoints in this way are demonstrated in Section 3.3.2. Although the system is based upon hand and arm keypoints, for OpenPose to locate hand keypoints, the upper body must be captured within the image. Upper body keypoints are also used to normalise the size and location of keypoints. Each pose is reduced to 8 upper body keypoints (wrists, elbows, shoulders, neck, and nose) and 42 hand keypoints (palms and 4 keypoints in each finger and thumb), ${P}_{f}=\{{j}_{f,1},\dots {,j}_{f,50}\}$ where ${j}_{f,i}=\left({x}_{f,i},{y}_{f,i}\right)$ are the co-ordinates of the *i*^th^ keypoint in frame *f*. When a body keypoint is occluded, OpenPose records the keypoint as missing. Conversely, all the hand keypoints are returned, even when the hand is occluded, if the wrist is detected. Missing values are imputed using the median location of keypoints in neighbouring frames.

#### Creating a Scale-Normalised Skeletal Pose

Each frame, *f*, is pre-processed to create a scale-normalised skeletal pose which is invariant to camera distance and participant size. The keypoint positions are translated so that the neck keypoint becomes the origin and scaled by dividing by the shoulder width, ${d}_{f}$, resulting in the scale-normalised skeletal pose, $\check{P}=\{\check{\jmath}_1,\dots{,\check{\jmath}}_{50}\}$:

1 $${d}_{f}=\sqrt{{\left({x}_{f,\mathrm{left}\_\mathrm{sh}}-{x}_{f,\mathrm{right}\_\mathrm{sh}}\right)}^{2}+{\left({y}_{f,\mathrm{left}\_\mathrm{sh}}-{y}_{f,\mathrm{right}\_\mathrm{sh}}\right)}^{2}}$$

2 $${\check{\jmath}}_{f,i}=\left(\frac{{x}_{f,i}-{x}_{f,\mathrm{neck}}}{{d}_{f}},\frac{{y}_{f,i}-{y}_{f,\mathrm{neck}}}{{d}_{f}}\right)$$

where ${\check{\jmath}}_{f,i}=({\check{x}}_{f,i},{\check{y}}_{f,i})$ and _*sh* is shoulder keypoint location.

A Savitzky-Golay filter (with polynomial order three and window length 17) is applied to each keypoint to smooth the data and remove noise. A Savitzky-Golay filter was selected due to its ability to smooth noisy signals with large frequency spans whilst maintaining the shape and height of the waveform peaks [37] that are indicative of agitated movement.

During a single-handed action, it is assumed that the hand that moves the most is performing the action. The hand with the most wrist movement is described as the dominant hand. Where necessary, skeleton sequences are reflected so that the right hand always appears to be the dominant hand. The dominant hand is deemed to be the wrist with the largest path distance during a sequence. Path distance is calculated for each wrist in a 180-frame sequence as:

3 $$\mathrm{path\:distance}={\sum }_{f=1}^{179}\mathrm{max}\left(\sqrt{{\left({x}_{f,\mathrm{wrist}}-{x}_{f+1,\mathrm{wrist}}\right)}^{2}+{\left({y}_{f,\mathrm{wrist}}-{y}_{f+1,\mathrm{wrist}}\right)}^{2}}\right)$$

where ${x}_{f,\mathrm{wrist}}$ is the *x* co-ordinate of the chosen wrist of frame *f*. The dominant hand is identified, and where necessary, the hands are reflected so that the right hand appears dominant. Finally, hand, wrist and elbow keypoints are re-centred around the midpoint between the wrists, resulting in the scale-normalised skeletal arm and hand pose ${\widehat P}_f=\left\{{\widehat j}_{f,1},\dots,{\widehat j}_{f,46}\right\}$ where:

4 $${\widehat j}_{f,i}=\left({\check{x}_{f,i}-\frac{\left(\check{x}_{f,\mathrm{right}\_\mathrm{wr}}+\check{x}_{f,\mathrm{left}\_\mathrm{wr}}\right)}2,\check{y}}_{f,i}-\frac{\left(\check{y}_{f,\mathrm{right}\_\mathrm{wr}}+\check{y}_{f,\mathrm{left}\_\mathrm{wr}}\right)}2\right)$$

and ${\check{x}}_{f,\mathrm{left}\_\mathrm{wr}}$ is the *x*-coordinate of the left wrist in frame f.

#### Locating the Centre of the Hand Using Hand Centroids

**Fig. 3:**
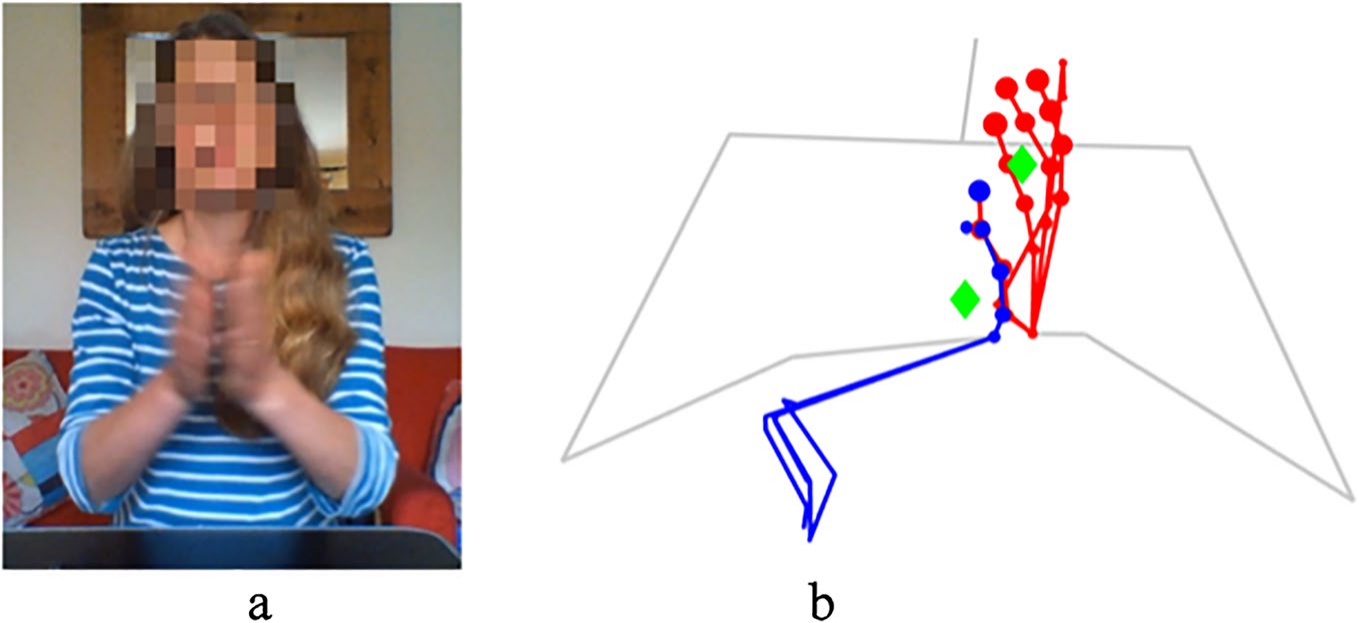
**b** The predicted hand pose of the subject in image **a**. Confidence scores are represented by the size of node at each keypoint (the larger the node, the higher the confidence score); the body pose has been faded. Whilst the predicted location of the left hand, plotted in red, appears relatively accurate and has correspondingly high confidence scores; the right hand, plotted in blue, contains many erroneous keypoints with low confidence scores. By using the confidence scores to weight predicted keypoint locations, hand centroids provide an accurate estimation of the location of hand centres

Analysing the pattern of a single keypoint or body part can enable the tracking of repetitive body movements [12, 23, 38]. It can, however, be unreliable to track hand movement with finger keypoints, as predicted hand keypoint locations are frequently erroneous due to fingers being hidden from the camera’s view. Hand centroids are calculated from the average of all hand keypoint locations, with each keypoint weighted according to the likelihood that a keypoint’s predicted location is accurate. For each keypoint, ${j}_{i}$, OpenPose provides a score ${c}_{i}$ in the range [0,1] based upon the confidence of its prediction. Figure 3b shows the OpenPose confidence scores represented by the size of the node at each keypoint. The hand centroid, shown in green, is estimated using the location $\left({x}_{i},{y}_{i}\right)$ and confidence score *c*_*i*_ of each the 21 keypoints for each hand:

5 $$\mathrm h\mathrm a\mathrm n\mathrm d\:\mathrm c\mathrm e\mathrm n\mathrm t\mathrm r\mathrm o\mathrm i\mathrm d=\frac1{\sum_{i=1}^{21}c_i}\left({\textstyle\sum_{j=1}^{21}}c_ix_i,{\textstyle\sum_{j=1}^{21}}c_iy_i\right)$$

### Classification of the Type of Repetitive Hand Movement

The most straightforward approach for classifying movement is to reduce each sequence to a single feature vector and apply a vector classifier. Sequences of skeletal poses have been summarised into vectors using bag of visual words [39] and temporal pyramids [40]. Vector classification has also been used for classifying agitation from accelerometer data [6]. However, informative features may be lost during vectorisation. Recurrent neural networks provide enable sequential classification without information loss and have been used for skeleton-based activity recognition [9, 41]. However, RNNs can require large amounts of training data. Moreover, whilst the ability to analyse sequential input is useful for activity recognition tasks, it may not be as beneficial in recognising repetitive movements of varying intensities. Naive Bayes nearest neighbour (NBNN) [42] measures the similarity between frames in sequences without considering the temporal order of frames, or the frequency of similar frames. The NBNN algorithm for sequence classification [43]:

Given an unlabelled sequence of *n* frames, *S* = $\left\{{\widehat P}_1,\dots,{\widehat P}_n\right\},$ each observation is matched with its nearest neighbour from each class of the training data. NBNN classifies a sequence by summing the distances between every frame and its nearest neighbour in each class. The predicted class c^*^ is the class with the minimum total distance between observations and nearest neighbours:

6 $$c^\ast={\mathrm{arg\:min}}_c{\textstyle\sum_i^n}\Arrowvert{\widehat P}_i-{NN}_c\lbrack{\widehat P}_i\rbrack\Arrowvert$$

where *NN*_*c*_ is the nearest neighbour of ${\widehat P}_i$ in class *c*. NBNN has been widely used for keypoint based activity recognition [39, 43, 44]. As an instance-based learner, however, NBNN can be computationally expensive for long sequences.

#### Selection of Discriminative Poses

**Fig. 4:**
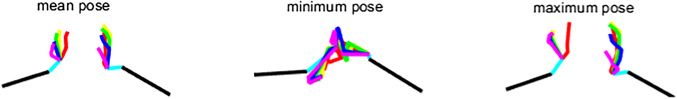
*Discriminative poses* consist of mean pose, the average of all hand poses in the sequence; minimum pose where hands are closest together; and maximum pose where hands are furthest apart

In this section, we describe a method to reduce the computational cost of using NBNN to classify repetitive movements which uses the *discriminative poses* that we introduced in an earlier publication [45]. Reducing a sequence to a single *mean pose* is a simple but effective way to classify sequences of repetitive movement. The *mean pose* is the mean position of each keypoint, *i*, over the 6-s (180 frame) sequence:

7 $${\mathrm{mean\:joint}}_{i}= \left(\frac{\sum_{f=1}^{180}{x}_{f,i} , \sum_{f=1}^{180}{y}_{f,i} }{180}\right)$$

where $\left({x}_{f,i},{y}_{f,i}\right)$ is the *i*^th^ keypoint in the *f*^th^ frame of the sequence. Additional informative poses are created to add dynamic information: the *maximum pose* of a sequence is the pose in which the hand centroids are furthest apart, whilst the *minimum pose* of a sequence is the pose in which hand centroids are closest together. Hence, a sequence of repetitive hand movements can be summarised by *discriminative poses* consisting of *mean, maximum* and *minimum poses*, as shown in Fig. 4. To add rigour, each 6-s sequence is divided into three 2-s (60 frames) sub-sequences, from which *mean*, *maximum*, and *minimum poses* are obtained; 60 frames is the smallest window in this dataset that always contains movement. Thus, each sequence is described by *discriminative poses*, consisting of nine unique poses, *D*_*i*_.

For NBNN classification, the class is determined as:

8 $$c^\ast={\mathrm{arg\:min}}_c{\textstyle\sum_i^9}\Arrowvert D_i-{NN}_c\lbrack D_i\rbrack\Arrowvert$$

The nearest neighbours for *mean*, *maximum*, and *minimum pose* are selected from within the same type of pose.

#### Accuracy for Classifying the Type of Repetitive Hand Movement

Three different cross-validation protocols were used with sequences from the training dataset to assess the effectiveness of using NBNN with discriminative poses for classifying the type of repetitive hand movements. To establish the overall effectiveness of our models, four-fold cross-validation is employed (Test 1). The ability of the model to generalise to different intensities is investigated using four-fold cross-intensity validation (Test 2). Both Test 1 and Test 2 training sets include data from test subjects. Finally, cross-subject validation is used to evaluate the model’s ability to generalise to new people (Test 3). The average percentage classification accuracy and standard deviation (in brackets) across all the folds of each test are reported in Tables 1, 2, and 3. The highest accuracy for each test is highlighted in bold.

**Table 1:** Comparison of sequence classifiers for classifying the type of repetitive hand movement

	NBNN: All frames Average % Accuracy	NBNN: Discriminative poses Average % accuracy	LSTM: All frames Average % accuracy
Test 1 (four-fold cross-validation)	98.50 (0.3)	**98.67** (0.27)	93.50 (0.8)
Test 2 (four-fold cross-intensity validation)	98.25 (1.1)	**98.17** (0.43)	89.83 (3.3)
Test 3 (15-fold cross-subject validation)	74.58 (11.9)	**76.17** (11.2)	71.08 (17.2)

**Table 2:** Comparison of vector classifiers for classifying the type of repetitive hand movement

Vector classifier (Test 3)	Average accuracy (%)
Linear SVM	**72.67** (14.6)
Random forest (10 trees)	68.50 (13.8)
Linear discriminatory analysis	67.58 (16.9)
Naive Bayes	71.33 (14.3)
Neural network (2 hidden layers)	64.25 (16.4)
K Nearest Neighbours (k = 5)	64.67 (16.3)

**Table 3:** Comparison of keypoints used for classifying the type of repetitive hand movement

NBNN: Discriminative poses (Test 3)	Average accuracy (%)
Hand and arm	**76.17** (11.2)
Body only	38.92 (10.9)
Hand only	60.83 (13.8)

Three sequence classification approaches were compared: the original NBNN using all 180 frames of the sequence (NBNN: All frames), our proposed approach using NBNN with 9 discriminative poses (NBNN: Discriminative Poses), and a LSTM using all 180 frames of the sequence (LSTM: All frames). A single layer, bi-directional LSTM with an “Adam” optimiser, 100 hidden layers, and a learning rate of 0.001 was used [46]. The average percentage classification accuracy and standard deviation (in brackets) across all the folds of each test are reported in Table 1; with the highest accuracy for each test highlighted in bold. Results show that NBNN with *discriminative poses* obtains the highest accuracy for all validation protocols. Moreover, using *discriminative poses* reduces the NBNN classification time by a factor of 1000, making them suitable for real-world applications. The high levels of accuracy obtained using NBNN with discriminative poses for Test 1 (98.7%) demonstrate the effectiveness of *discriminative poses* for recognising repetitive movements, whilst the 98.2% accuracy obtain for Test 2 establishes that *discriminative poses* can be used to recognise repetitive movement even when trained using movements with intensities that are different from the test data. Cross-subject (Test 3) accuracy varied between 54 and 96%, with an average accuracy of 76.2%, indicating that frequently the approach is unable to generalise to new subjects. This suggests the benefit of including some end-user information in the training dataset.

To demonstrate the advantage of using NBNN in comparison to vector classifiers, the nine *discriminative poses* were concatenated into a single vector and reduced using PCA to create a single vector contain 99% of the variance of the original sequence. Table 2 shows that whilst classification using linear SVM obtains the highest accuracy of all the vector classifiers, and was more accurate than using a LSTM, NBNN was the most accurate. Additional experiments demonstrate the advantages of using both hand and arm keypoints to recognise hand movement. Table 3 shows that models created using hand and arm keypoints obtain higher accuracies than using only upper body keypoints or only hand keypoints. As in Table 1, in Tables 2 and 3 the average percentage accuracy is reported for each experiment, with the standard deviation given in brackets. The highest accuracy for each test is highlighted in bold.

Having established that the type of hand movement can be automatically recognised from video-based keypoints, the next section presents an approach which makes use of the predicted type of movement to assess the intensity of the movement.

### Automatic Assessment of the Intensity of Movement

The intensity of a repetitive movement can be defined by the frequency of repetitions over a specified period. Step counting is a common application where repetitive movements are counted. Most step counters use accelerometers; however, video-based keypoints have been used for monitoring gait within a clinical setting [28]. Individual steps are identified using pattern matching approaches such as DTW [10] and HMM [10, 27], or peak detection [10, 11, 28]. Another application is where the intensity of repetitive movements is assessed in the finger tap test; a clinical test was used for assessing bradykinesia. Finger tapping has been captured using electromagnetic sensors [30, 31] or video-based keypoints [12, 29]. The rate of movement has been assessed using evolutionary algorithms [30, 31], discrete wavelet transformations [29], and peak detection [12]. Instead of counting the exact number of finger taps, all the systems use a clinical scale to classify the extent of bradykinesia.

Unlike walking and finger tapping, which contain little inter-subject variation, repetitive hand movements can be performed in many ways. Due to intra-class and inter-subject variation, repetitive hand movements often only become recognisable at key frames in the sequence — as demonstrated by the effectiveness of *discriminative poses*. This makes pattern matching approaches unsuitable for assessing the intensity of repetitive movements. Peak detection, however, relies on identifying the key frames in a signal, extrema denoting where keypoints are closest together or furthest apart, making peak detection suitable for detecting the intensity of repetitive movements with wide variations.

#### Peak Detection

**Fig. 5:**
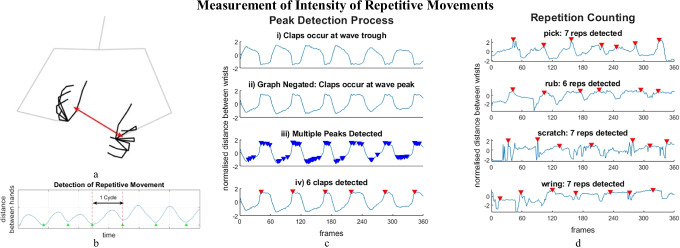
**a** The distance between hands can be used to measure the intensity of movement (unused body keypoints are shown faded). **b** A single 1D signal is used to represent the movement of clapping. Green arrows mark signal troughs where hands are closest together. **c** The process of detecting repetition using peak detection involves negating the signal and setting hyperparameters to identify distinct peaks. **d** Peak detection can be used to count repetitions from all types of repetitive movements

To capture the temporal dynamics of a movement, sequences must contain several full repetitions of the movement. Each 30-s sequence from our original training data is redivided into four 12-s windows with a 5-s overlap, discarding 1½ s at the start and end of each sequence. Repetitive movement can be represented as a one-dimensional trajectory enabling periodic patterns to be identified for repetitive movements [10–12, 28]. Representing movement by a single parameter reduces variation between how actions are demonstrated whilst retaining key characteristics of the movement. Peak detection is used to count repetitions in the signal’s pattern for all the hand movements in the experiment. Figure 5a–c shows the process for clapping. The distance between hands plotted as a time-series signal, as illustrated in Fig. 5a and b. By assuming that there is one local minimum for each clap, repetitions can be counted. Figure 5c shows the process of locating local minima. Firstly, the original signal is negated so that the clap occurs at the wave’s peak. Since hand movement is not always smooth, multiple non-clap local peaks may be detected. The algorithm is optimised by tuning hyperparameters to ignore unrealistic peaks. Three hyperparameters are used: *minimum peak prominence* may be set to ensure that only distinct peaks are selected; *minimum peak height* may be set to disregard peaks below a threshold; *minimum peak distance* may be set to disregard peaks that are too close together. Whilst other approaches have based hyperparameters upon physiological evidence of the specific body movements, [10, 12], the variations within each type of hand movement means that it is not evident in advance how hyperparameters should be set. Therefore, a grid search is used to tune the hyperparameters with the training data. The search areas, given in Table 4, are based upon the normalised distance between hands. Hyperparameters are tuned separately for the four distance parameters and five repetitive hand movements. Figure 5d illustrates that this approach can be used for all five types of repetitive hand movements.

**Table 4:** Grid search parameters for tuning peak detection algorithm

Parameter	Units	Search area
Minimum peak prominence	Distance parameter	0:0.2:2
Minimum peak distance	Frames	10:20
Minimum peak height	Distance parameter	− 0.5:0.1:0.5

#### Comparison of Distance Measure

**Fig. 6:**
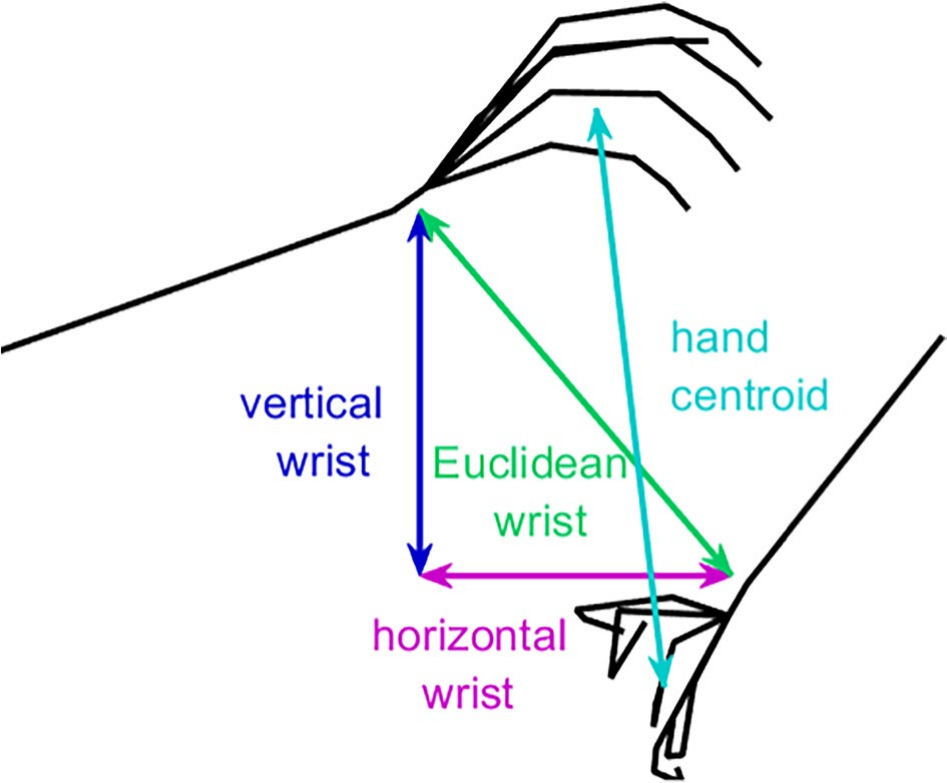
Distance measures for assessing hand movement

In Fig. 5, clapping is modelled using the *Euclidean distance between hand centroids*. However, alternative distance measures could have been used. Moreover, the most appropriate distance measure for each type of movement may differ. As illustrated in Fig. 6, three other sets of distance measures were considered: *Euclidean distance between wrists*, *vertical distance between wrists*, and *horizontal distance between wrists*. Distances for each sequence were normalised in the range [0,1], enabling comparisons between sequences where the subjects moved their hands with different levels of mobility.

#### Measures of Accuracy

Intensity is measured according to the number of repetitions of a movement within a sequence. Two different measures of accuracy are used. *Range intensity* determines whether the category of intensity of a sequence has been correctly predicted. Repetitions are considered to be correctly counted if the number of detected repetitions falls between a range whose boundaries are determined by the metronome’s speed.

9 $$\mathrm{range\:accuracy}=\left(\frac{\mathrm{number\:of\:intensities\:correctly\:predicted\:from\:within\:a\:globally\:defined\:range}}{\mathrm{total\:number\:of\:sequences}}\right)\times 100$$

*Personalised intensity* assesses the ability of the model to differentiate between a subject’s movement at different speeds. Personalised boundaries are based upon the average number of repetitions detected at each intensity and are reset for each activity and participant. An intensity is detected correctly when the number of repetitions detected falls between two boundaries; the lower boundary is the mean number of repetitions detected for sequences for which the correct intensity is one category slower than the correct intensity of the examples being considered; the higher boundary is the mean number of repetitions detected for sequences for which the correct intensity is one category faster than the correct intensity of the examples being considered. Criteria for determining the range and personalised boundaries are set out in Table 5.

**Table 5:** Boundaries for the number of movements detected at each level of Intensity

Category of intensity	Range boundaries	Personalised boundaries
Slow	Reps ≤ 8	Reps < mean reps for *medium*
Medium	8 < reps ≤ 12	Mean reps for *slow* < reps < mean reps for *medium fast*
Medium fast (MedFast)	12 < reps ≤ 16	Mean reps for *medium* < reps < mean reps for *fast*
Fast	Reps > 16	Mean reps for *medium fast* < reps

Reps = number of repetitions of a movement detected within a 12-second sequence. Mean reps = mean number of repetitions detected for an individual participant demonstrating a movement at an intensity

10 $$\mathrm{personalised\:accuracy}= \left(\frac{\mathrm{number\:of\:intensities\:correctly\:predicted\:from\:within\:a\:personalised\:range}}{\mathrm{total\:number\:of\:sequences}}\right)\times 100$$

Whilst range accuracy is the more stringent measure of the accuracy, personalised accuracy is sufficient for identifying changes in the intensity of a movement, and so is ideal for monitoring agitation of an individual. Range accuracy is used during the grid search to set the hyperparameters, whilst personalised accuracy is reported in the test data results.

#### Tuning of Hyperparameters and Selection of Distance Measures

**Fig. 7:**
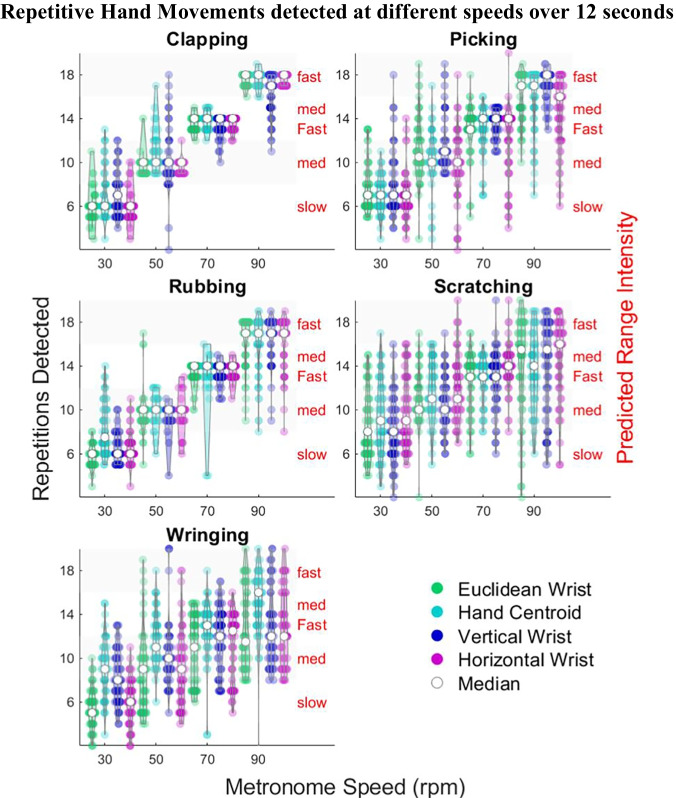
Violin plots illustrating the number of repetitive hand movements detected at the different intensities using each distance parameters. Repetitions detected are shown on the left axis, and the categories of range intensities (slow, medium, medium-fast, and fast) are on the right

Hyperparameters were tuned by a grid search separately for each class of hand movement and distance parameter from the training data. Table 6 reports the optimally tuned hyperparameters with both the range and personalised classification accuracies for each type of repetitive hand movement. Whilst high range accuracies were obtained for clapping and rubbing, the accuracy for detecting the intensity of wringing and scratching was much lower. Personalised accuracies were higher than range accuracies for all hand movement types, indicating that although counting the exact number of repetitions can be problematic, differences in the intensity of an individual’s behaviour can be detected for all types of hand movement. The *hand centroid* and *horizontal wrist* parameters did not perform best for any of the five types of repetitive hand movement and so were not used with the test data in the final model. The violin plots in Fig. 7 provide a visual comparison of the effectiveness of each distance parameter for the different types of repetitive hand movement. The plots show the number of detected repetitions increasing as the true intensity increases, demonstrating that differences between the speed of repetitive movements have been detected, illustrating that high personalised accuracies can be achieved for sequences with low range accuracy.

**Table 6:** Tuned peak detection hyperparameters for proposed model determined using grid search with range accuracy

	Distance parameter	Hyperparameter: Minimum peak			% Accuracy	
		Prominence	Distance	Height	Range	Personalised
Clap	Euclidean wrist	0.8	10	-0.65	97.1	99.2
Pick	Vertical wrist	0.5	11	-0.2	75	94.2
Rub	Vertical wrist	0.6	10	-0.5	88.3	94.6
Scratch	Euclidean wrist	0.4	10	-0.5	58.3	72.5
Wring	Euclidean wrist	0.4	10	-0.4	48.8	86.7

## Results

The proposed system, illustrated by the pipeline in Fig. 1, is completely automated, first classifying the type of movement, then assessing changes in the intensity of repetitive hand movements. A test dataset of 400 12-s sequences was created from sequences demonstrated by the five reserved test participants using the approach described in Section 3.4.1 (resulting in four sequences of five repetitive movements at four different intensities from each participant).

### Assessment of the Type and Intensity of unknown Repetitive Hand Movements

**Fig. 8:**
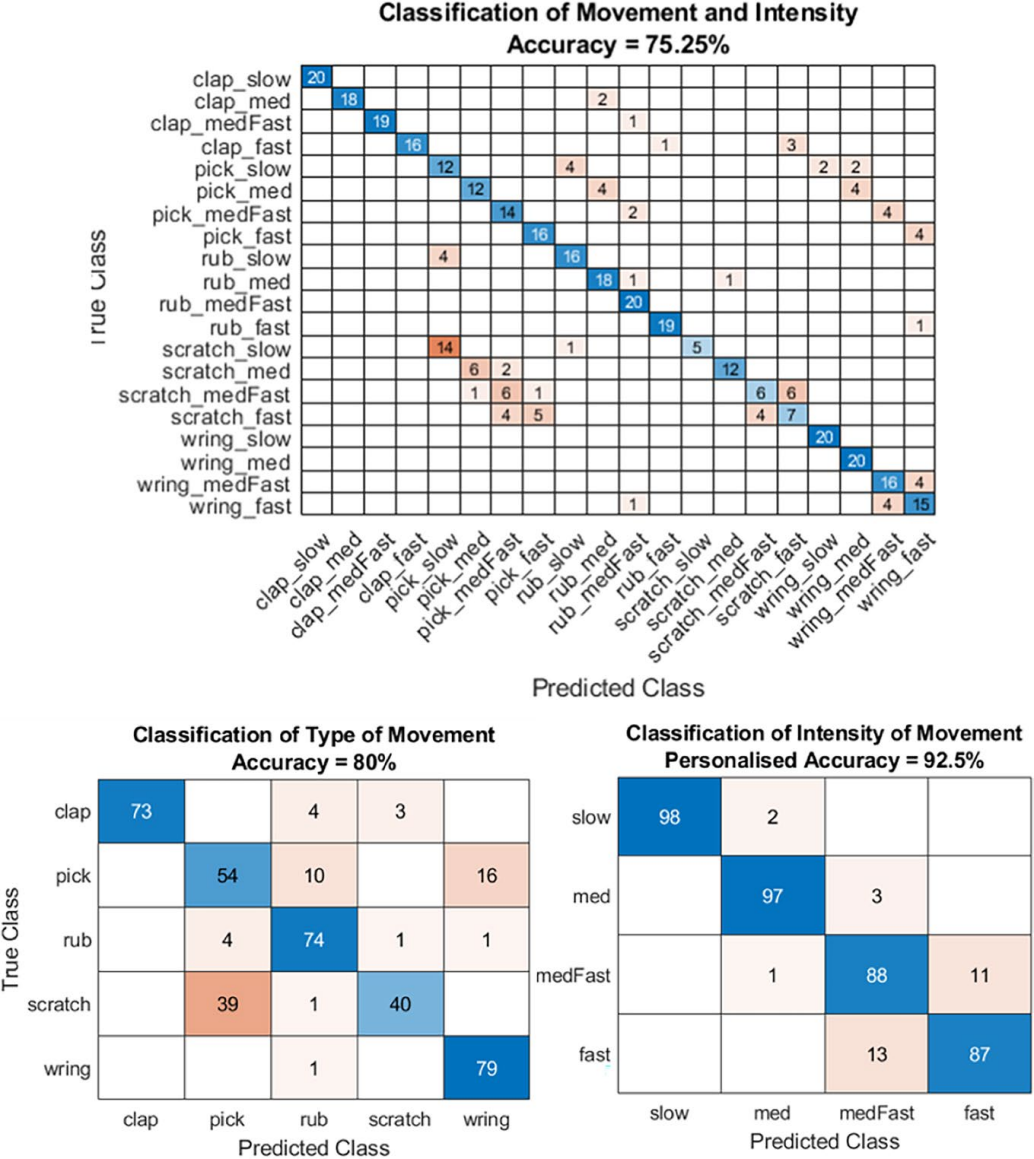
Results from the unseen test dataset using parameters learnt from the training and validation dataset. Top: An overall personalised accuracy of 75.25% was obtained for classification of type and intensity of movements. 80% of the actions and 92.5% of the speeds were classified correctly

Using signals created from the distance measure determined by the predicted type of repetitive hand movement, peak detection was used to assess changes in intensity. Personalised accuracy was used to measure the ability of the proposed approach to differentiate between movements of different intensities. Both the type and the intensity of movement were classified correctly in 75.25% of the test sequences. The type of movement was classified correctly in 80.0% of sequences, whilst the intensity of movement was classified correctly in 92.5% of sequences. Results for each stage of the system are presented in the confusion matrices in Fig. 8.

### Effectiveness of using the predicted type of movement to assess the Intensity of Movement

As the type of movement was predicted incorrectly in 20% of the sequences, we explore the importance of first predicting the type of movement before assessing intensity by comparing the proposed model with models built under three different scenarios. In the first scenario, we assume that the type of hand movement was known. The class-specific parameters, tuned on the training data and given in Table 6, were assigned to each sequence according to the ground truth labels. In the second scenario, we assume that the type of movement is not relevant for measuring intensity, creating a generic model suitable for all types of hand movement. The classification step is omitted, and Euclidean wrist distance parameter used, chosen as it embodies both horizontal and vertical wrist movements. Hyperparameters are set following a grid search of the entire training dataset. In the final scenario we assess whether instead of using class-specific parameters, more effective parameters can be learnt by clustering similar types of movement. The sequences of the nine discriminative poses from the training dataset were concatenated into a single feature vector (of length 828) and partitioned into five clusters using k-means clustering with the squared Euclidean distance metric. A grid search of the training data was used to optimise the distance parameter and hyperparameters for each cluster. Vectorised sequences of discriminative poses from the test dataset are assigned to clusters using nearest centroid classification. Cluster specific parameters are used to classify the intensity of repetitive hand movement in sequences from the test datasets.

The accuracy of our proposed model and the three models built under the different scenarios are reported in Table 7. Whilst similar range and personalised accuracies were achieved for all four models, the proposed model obtained the highest range and personalised accuracies. Of the remaining three scenarios, the generic model performed the best, misclassifying only seven more sequences than our proposed approach. The slightly higher accuracy achieved using the predicted class labels rather than the ground-truth labels may suggest that the characteristics of the movement, summarised by discriminative poses, are more informative for measuring intensity than the actual ground-truth labels themselves. However, the clustered model, performed less well than the proposed approach, suggesting that the type of movement is relevant to assessing intensity.

**Table 7:** Accuracy of models assessing the intensity of repetitive hand movements, the highest accuracy for each model is highlighted in bold

Models	% range accuracy	% personalised accuracy
Proposed	**79.25**	**92.50**
Known	76.75	88.50
Generic	76.75	90.75
Cluster	77.75	90.00

## Conclusion

We have proposed a model for efficiently monitoring the type and intensity of repetitive hand movements, based upon *discriminative poses* and peak detection. Our model was able to correctly identify both the type and intensity of repetitive hand movements in 75% of the sequences performed by unseen subjects. We have shown that despite relatively accuracy for classifying the type of movement (80%), first predicting the type of movement can aid assessment of the intensity of repetitive movements by enabling the selection of appropriate distance measures for measuring the intensity of movements.

The lower accuracy for recognising the type of movement obtained in the cross-subject tests (Test 3) than in the non-cross subject tests (Tests 1 and 2) suggests that classification of the type of movement could be improved by training the model with subject-specific data. Due to variations in how people perform different movements, the inclusion of person-specific observations in the training dataset can significantly improve recognition accuracy. Whilst the ability of an activity recognition model to generalise to new subjects is generally considered important for activity recognition, some researchers have argued that for a model to be robust in real-world applications, personalisation is essential [47]. As NBNN is non-parametric, personalising the algorithm simply requires carers to update the training dataset with user specific examples of behaviour. The approach of updating a model with user-specific data has been proposed by other agitation detection systems [7] and is particularly suitable for activity recognition models, such as for agitation monitoring, which have a single end user.

The experiments in this study have used healthy volunteers demonstrating repetitive hand movements indicative of agitation, where the speed of movement was prescribed by the researcher. Whilst the study has demonstrated that the type and intensity of repetitive movement can be automatically detected, further studies using data captured from people who experiencing agitation are necessary to confirm whether the system is suitable for use within a care setting. However, the high levels of personalised accuracy achieved for assessing the intensity of repetitive behaviours suggest that the system is likely to be able to assess changes in behaviours across a wide variety of different behaviours. This study has shown the potential for video-based systems to supplement existing care of people living with dementia by automatically monitoring the type and intensity of agitated episodes. In this study, the first to focus on agitated hand movements, we have concentrated on recognising and assessing the intensity of repetitive movements. Future work will explore the detection of repetitive hand movements in order to automatically differentiate between agitated movements and normal behaviours. Whilst this study used data from healthy participants, the results have shown the value in additional studies using people living with dementia.
